# Assessment of Age From Sternal Fusion: A Radiographic Autopsy-Based Study in North India

**DOI:** 10.7759/cureus.92253

**Published:** 2025-09-13

**Authors:** Arushi Verma, Shailesh V Parate, Raviprakash Meshram, Vikas Vaibhav, R Sivasankary, Ashish R Bhute, Anubhuti Tyagi

**Affiliations:** 1 Forensic Medicine and Toxicology, All India Institute of Medical Sciences, Rishikesh, Rishikesh, IND; 2 Forensic Medicine and Toxicology, All India Institute of Medical Sciences, Vijaypur, Jammu, IND

**Keywords:** age estimation, forensic anthropology, manubrio-sternal junction, north india, sterno-xiphoidal junction, sternum

## Abstract

Age estimation plays a crucial role in forensic identification, especially in cases involving dismembered or fragmented human remains. The sternum bone, often retrievable even in fragmented remains, holds potential value in age estimation. The present autopsy-based study aimed to estimate the age of fusion of the manubrio-sternal (M-S) and sterno-xiphoidal (S-X) junctions among males and females aged 14 years and above in the North Indian population using the radiographic (X-ray) method and to assess its applicability in forensic age estimation.

A total of 195 sterna (165 males, 30 females) were examined for the fusion status of M-S and S-X junctions during medico-legal autopsies at AIIMS Rishikesh. Radiographic examination (X-ray) was performed, and the fusion was categorized as either fused or unfused. The age of the deceased was confirmed via official documents. Fusion at the M-S junction was observed in 23.6% (n=46) of cases and at the S-X junction in 34.4% (n=67) of cases. The earliest fusion at both junctions occurred at 19 years in males and 40 years in females. A moderate association was noted between age and M-S junction fusion in females (bias-corrected Cramer's V = 0.36), with receiver operating characteristic (ROC) analysis showing 100% sensitivity and 71% specificity at 40 years. In contrast, S-X junction fusion in males demonstrated a moderate association (bias-corrected Cramer's V = 0.35), with ROC analysis showing 81% sensitivity and 57% specificity at 34 years. No significant associations were observed between fusion of the M-S junction and age among males and between fusion of the S-X junction and age among females.

These findings suggest that fusion of the M-S junction may help in age estimation in females, while fusion of the S-X junction could serve as a marker in males. However, due to inter-individual variability and limited female representation in our study sample, further studies with larger and more balanced samples are needed for confirmation of the findings of our study.

## Introduction

Identification of individuals holds significant importance across administrative, ethical, and legal domains. In medico-legal cases involving unidentified bodies or human remains, one of the primary goals of an autopsy is to formulate the biological profile of the individual to aid in identification [1]. Given the variability across populations, such profiles must be adapted using region-specific standards. Among the critical components of a biological profile - age, sex, stature, and ancestry - age estimation plays a pivotal role, especially when matching unidentified human remains with missing persons [1].

When bodily remains are intact, age assessment is relatively straightforward. However, in cases involving advanced decomposition or severe damage, dental and skeletal analysis becomes essential. Various skeletal markers, including cranial suture closure and the morphology of bones such as the ilium and clavicle, and dental development, have been studied extensively [2-9]. Despite advancements in the field, estimating age in adults remains a challenge due to individual variability [1].

The sternum, often retrievable even in fragmented remains, is less explored compared to other bones. Nevertheless, previous studies suggest its potential value in age estimation. Anatomically, the sternum comprises three parts - the manubrium, the mesosternum (body), and the xiphoid process - developing through endochondral ossification. The fusion status of the manubrio-sternal (M-S) and sterno-xiphoidal (S-X) junctions, assessed via visual or radiological methods, can prove to be useful for age estimation [10]. This study aims to evaluate the age of fusion at these junctions among males and females aged 14 years and above in the North Indian population using the radiographic (X-ray) method, and to assess its applicability in forensic age estimation.

## Materials and methods

Our study examined 195 sterna (165 males and 30 females) obtained from deceased individuals aged 14 years and above, belonging to North India, with legal proof of age and residence. The specimens were collected from all consecutive medico-legal autopsies fulfilling the inclusion criteria, conducted at the Department of Forensic Medicine and Toxicology, AIIMS Rishikesh, over an eight-month period (August 2022 to March 2023). Age was determined based on information from police inquest papers and verified against official identification documents, including Aadhaar cards and other legal records provided by legal heirs. Sterna with any fractures, congenital anomalies, or deceased lacking legal proof of age were excluded from the study. 

During the autopsy, an "I"-shaped incision was given, and the skin, subcutaneous tissue, fat, and underlying muscles were carefully reflected. The ribs were cut laterally at the costochondral junctions, and the sternum was detached by disarticulating both sternoclavicular joints.

**Figure 1:**
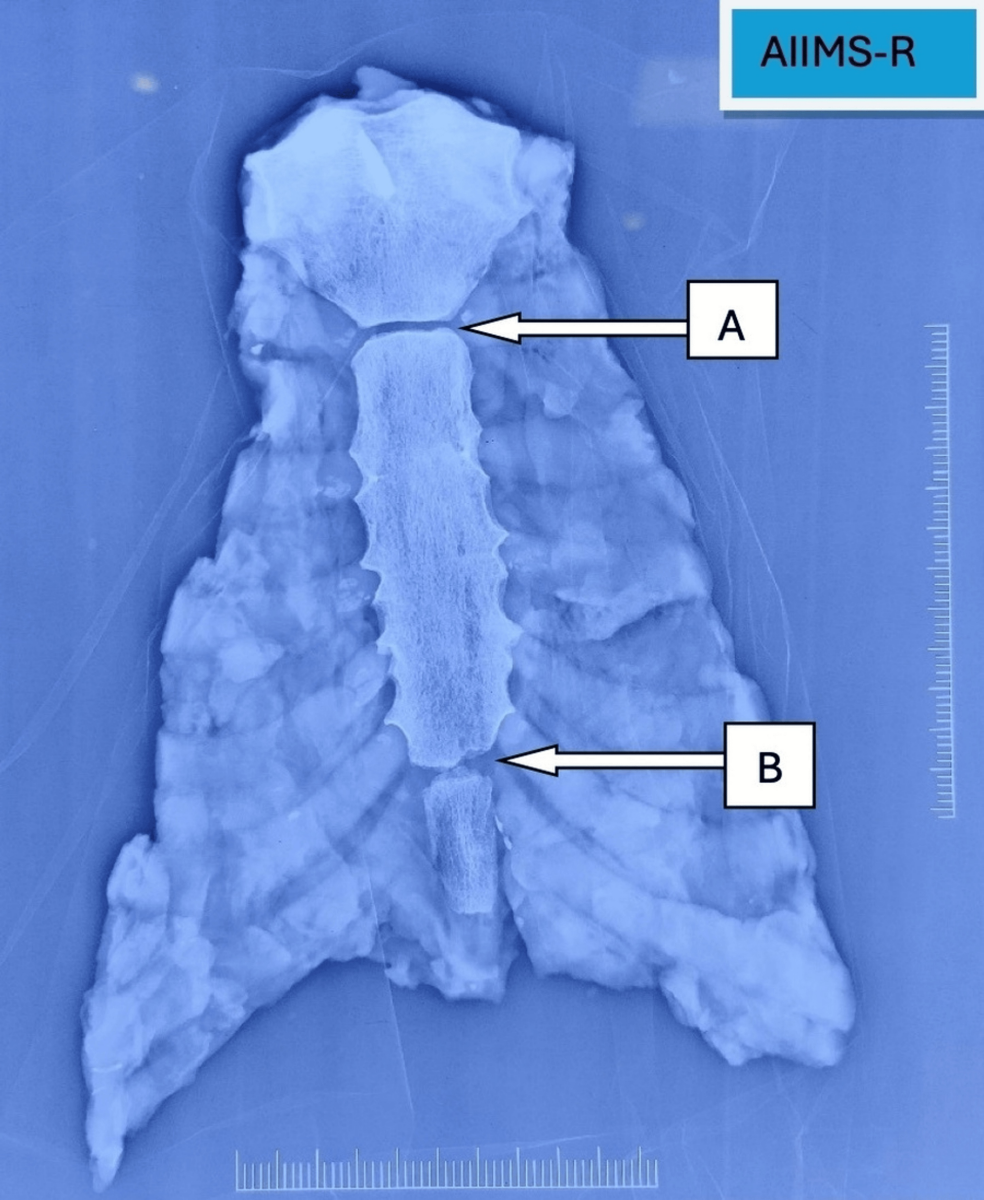
Radiological (X-ray) examination of sternum bone showing unfused M-S junction (A) and unfused S-X junction (B) M-S, manubrio-sternal; S-X, sterno-xiphoidal.

**Figure 2:**
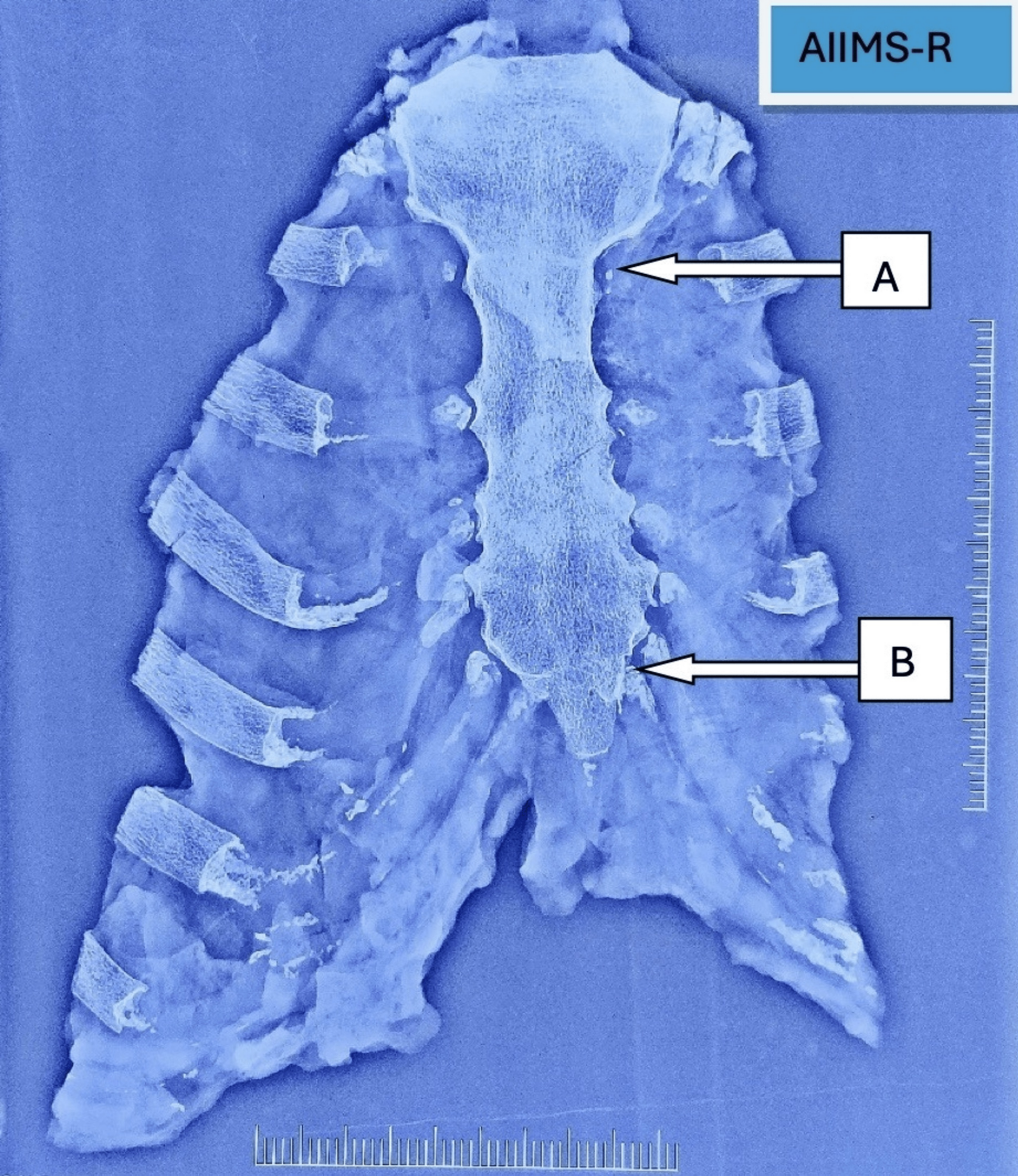
Radiological (X-ray) examination of sternum bone showing fused M-S junction (A) and fused S-X junction (B) M-S, manubrio-sternal; S-X, sterno-xiphoidal.

The excised sterna were cleaned of soft tissue, dried, and visually assessed for fusion status. Radiological examination (X-ray, anterior-posterior view) was then performed on the sternum using an X-ray machine "SKANMOBILE, Mobile X Ray Radiology system" (Skanray Technologies Pvt. Ltd, Mysore, India) with exposure parameters of 60 kVp and 16 mA and an image reader "FUJIFILM, FCR PROFECT CS Plus" (Fujifilm Corporation, Tokyo, Japan) to comment on the fusion status (Figures 1, 2). On analysis of radiographs, the fusion was graded into two categories: unfused and fused. Fusion was defined by complete obliteration of the joint space, whereas any remaining joint space was classified as unfused. The observer was blinded to the age of the deceased during evaluation. Data were analyzed using IBM SPSS (Statistical Package for the Social Sciences), Version 23.0 (IBM Corp., Armonk, NY). Descriptive statistics were used to describe the population characteristics of the sample and to show the prevalence of fusion throughout the sample. Appropriate statistical tests were used to determine if there was an association between age and fusion of the sternal junctions. ROC (receiver operating characteristic) curve analysis was performed to predict an individual’s age based on their fusion status. The level of significance was set at a standard p-value of  less than 0.05.

Ethical clearance was not required for the present study as it did not involve any living human participants, and the sternum was temporarily removed as a part of a routine medico-legal autopsy procedure conducted under statutory authority in India. Following removal, radiographic imaging was performed on the excised bone, which was subsequently replaced within the thoracic cavity before body reconstruction and handover to the legal heirs. Confidentiality of all identifying personal information was strictly maintained throughout the data collection and analysis process.

## Results

The total number of sternum studied in the present study was 195 [males: 165 (84.6%), females: 30 (15.4%)]. The age of the participants ranged from 15 to 85 years, with a mean age of 39 years.

In this study, 23.6% (n=46) of the sternum displayed fusion at the M-S junction and 34.4% (n=67) at the S-X junction. The mean age of the deceased with a fused M-S junction was 45 years (males: 43 years; females: 56 years), and with a fused S-X junction was 47 years (males: 47 years; females: 53 years). The earliest age at which fusion of the M-S junction was observed was found to be 19 years in males and 40 years in females, which was the same as that of the S-X junction. Non-fusion of the M-S junction was observed up to 82 years in males and 76 years in females, and that of the S-X junction was observed up to 85 years in males and up to 76 years in females.

**Figure 3:**
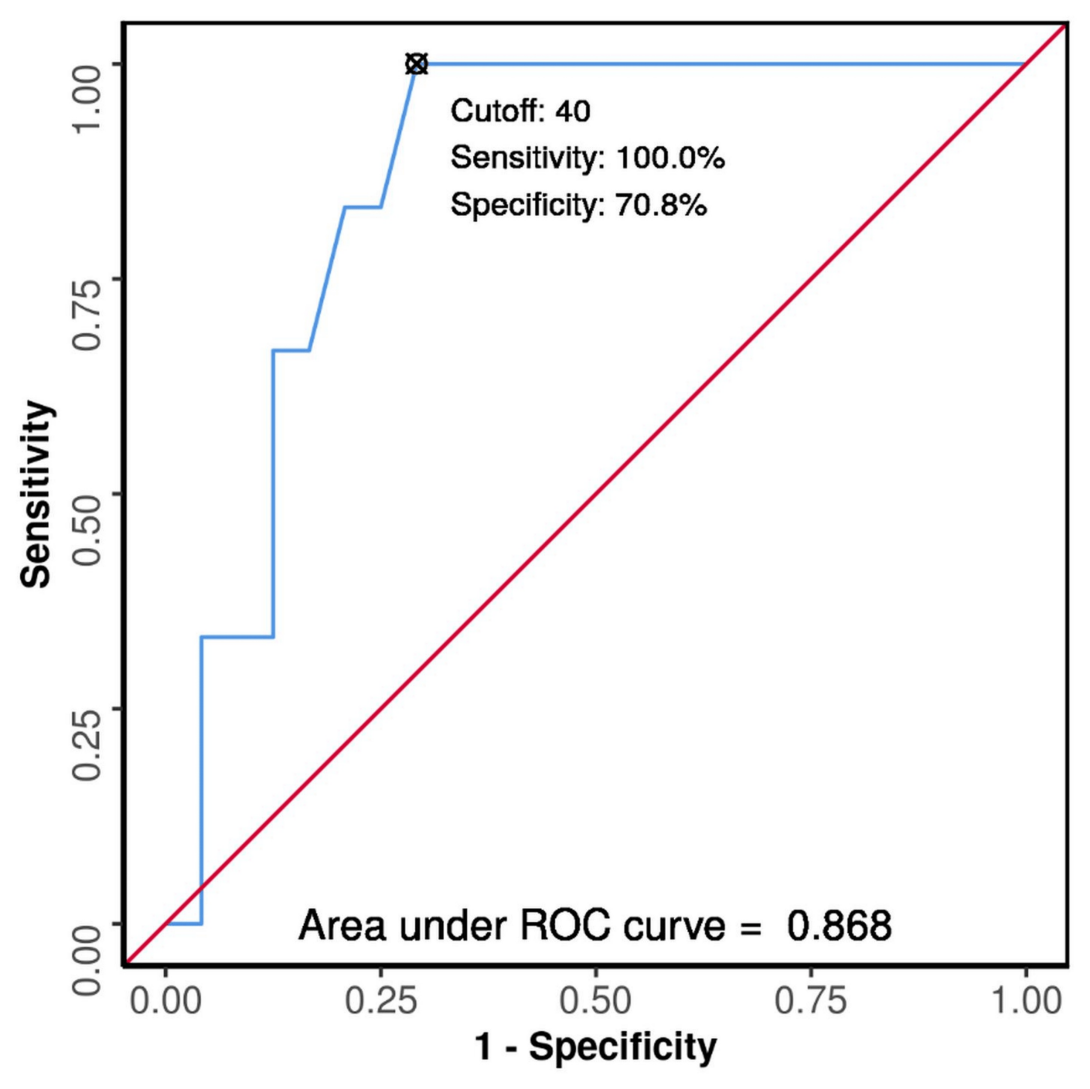
ROC curve analysis showing diagnostic performance of age (years) in predicting fusion of M-S junction in females (n=30) The AUROC for age (years) predicting fusion of the M-S junction in females was 0.868 (95% CI: 0.739-0.997), thus demonstrating good diagnostic performance. It was statistically significant (p = 0.006). M-S, manubrio-sternal; ROC, receiver operating characteristic; AUROC, area under the ROC curve.

Fisher’s exact test was used to explore the association between age and fusion of the M-S junction in both males and females. The results showed no significant difference between the fused and unfused groups with respect to the M-S junction in males (X^2^=7.094, p=0.354). Therefore, it was concluded that there is no/little association between age and fusion of the M-S junction in males (bias-corrected Cramer’s V = 0.02). However, in the case of females, the test results showed a significant difference between the fused and unfused groups with respect to the M-S junction (X^2^=10.000, p=0.043). Therefore, it was concluded that there is a moderate association between age and fusion of the M-S junction in females (bias-corrected Cramer’s V = 0.36) (Table 1). An ROC curve analysis showing diagnostic performance of age in predicting fusion of the M-S junction was performed in females, which revealed that the M-S junction was fused with a sensitivity of 100% (95% CI: 54-100) and a specificity of 71% (95% CI: 49-87) at a cutoff age of 40 years and above (Figure 3). However, in males, no statistically significant results were observed.

**Table 1:** Association between "age" and "fusion of M-S junction" among males and females M-S, manubrio-sternal.

Age (years)	Males			Females		
	Number	Fused (%)	Unfused (%)	Number	Fused (%)	Unfused (%)
10-19	6	2 (33.3%)	4 (66.7%)	2	0 (0%)	2 (100%)
20-29	43	8 (18.6%)	35 (81.4%)	12	0 (0%)	12 (100%)
30-39	44	10 (22.7%)	34 (77.3%)	3	0 (0%)	3 (100%)
40-49	35	7 (20%)	28 (80%)	5	2 (40%)	3 (60%)
50-59	22	6 (27.3%)	16 (72.7%)	4	2 (50%)	2 (50%)
60-69	7	4 (57.1%)	3 (42.9%)	2	1 (50%)	1 (50%)
70-79	4	1 (25%)	3 (75%)	2	1 (50%)	1 (50%)
80-89	4	2 (50%)	2 (50%)	0	0	0
Total	165	40 (24.2%)	125 (75.8%)	30	6 (20%)	24 (80%)

**Figure 4:**
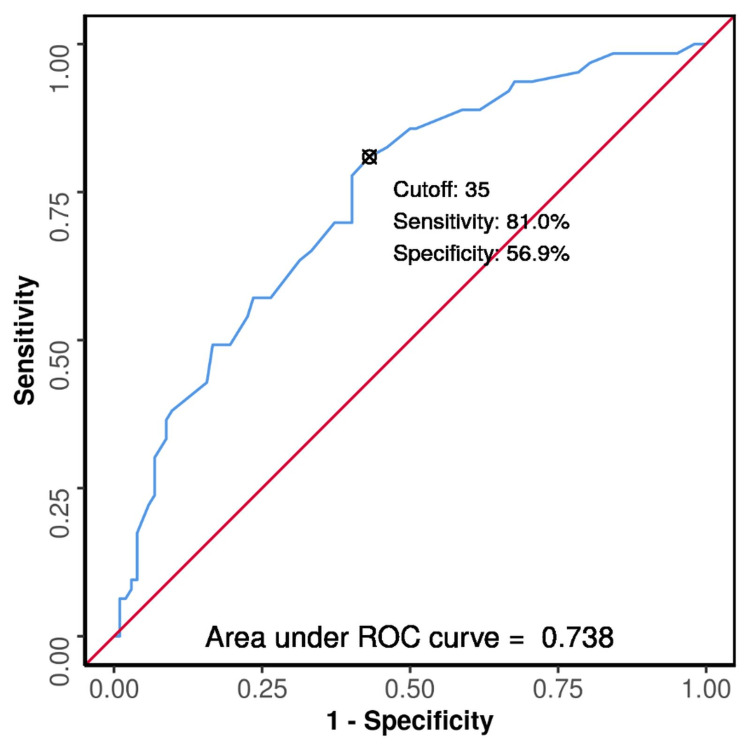
ROC curve analysis showing diagnostic performance of age (years) in predicting fusion of S-X junction in males (n=165) The AUROC for age (years) predicting S-X junction:fused vs S-X junction:unfused was 0.738 (95% CI: 0.661-0.815), thus demonstrating fair diagnostic performance. It was statistically significant (p≤0.001). S-X, sterno-xiphoidal; ROC, receiver operating characteristic; AUROC, area under the ROC curve.

Chi-squared test and Fisher’s exact test were used to explore the association between age and fusion of S-X in males and females, respectively. The results of the test showed that there was a significant difference between the fused and the unfused groups with respect to the S-X junction in males (X^2^=27.530, p=0.001). Therefore, it was concluded that there is a moderate association between age and fusion of the S-X junction in males (bias-corrected Cramer’s V=0.35). However, in the case of females, the results showed no significant difference between the fused and the unfused groups with respect to the S-X junction (X^2^=8.798, p=0.129). It was concluded that there was a low association between age and fusion of the S-X junction in females (bias-corrected Cramer’s V=0.3) (Table 2). An ROC curve analysis showing diagnostic performance of age in predicting fusion of the S-X junction was performed in males, which revealed that the S-X junction was fused with a sensitivity of 81% (95% CI: 69-90)and a specificity of 57% (95% CI: 47-67) at a cutoff age of 34 years and above (Figure 4). However, no statistically significant results were observed in females.

**Table 2:** Association between "age" and "fusion of S-X junction" among males and females S-X, sterno-xiphoidal.

Age (years)	Males			Females		
	Number of sterna	Fused (%)	Unfused (%)	Number of sterna	Fused (%)	Unfused (%)
10-19	6	1 (16.7%)	5 (83.3%)	2	0 (0%)	2 (100%)
20-29	43	6 (14%)	37 (86.0%)	12	0 (0%)	12 (100%)
30-39	44	16 (36.4%)	28 (63.6%)	3	0 (0%)	3 (100%)
40-49	35	15 (42.9%)	20 (57.1%)	5	2 (40%)	3 (60%)
50-59	22	14 (63.6%)	8 (36.4%)	4	1 (25%)	3 (75%)
60-69	7	6 (85.7%)	1 (14.3%)	2	0 (0%)	2 (100%)
70-79	4	2 (50%)	2 (50.0%)	2	1 (50%)	1 (50%)
80-89	4	3 (75%)	1 (25%)	0	0	0
Total	165	63 (38.2%)	102 (61.8%)	30	4 (13.3%)	26 (86.7%)

## Discussion

Historical literature, dating back to Coiter’s citations of Fallopius and Eustachius, recognized that fusion of the M-S and S-X joints parallels cranial suture fusion, often occurring beyond the age of 30 [11]. Ashley (1954) studied 1050 sterna radiographically and found that M-S and S-X fusion commonly occurs in individuals over 30, and he was the first to show that M-S fusion is not confined to old age. He categorized this fusion into primary (matrical) synostosis, resulting from early-life closure of the primary cartilaginous joint, and secondary (sclerotic) synostosis due to late-adulthood pathological closure of the secondary cartilaginous joint. As this joint contributes significantly to chest expansion and respiratory mechanics, early matrical fusion may plausibly impair pulmonary function and increase susceptibility to lung diseases such as tuberculosis. This insight prompted reevaluation of prior beliefs that fusion at the M-S joint was purely an age-related degenerative process [12]. Jit and Bakshi (1986) examined 980 sterna (772 male, 208 female) using visual and radiographic methods and demonstrated that the earliest age of fusion of the M-S junction was 21 years in both sexes, while that of the sterno‑xiphisternal junction was 18 years in males and 21 years in females, but either junction can remain unfused well into later years. Consequently, they concluded that sternal ossification after age 18-20 is too variable to reliably estimate age [13]. Sobhan K. Das (2005) proposed tentative age thresholds for estimating age based on the presence of fusion at the sternum junctions: 28 years for M-S, 32 years for S-X, and 36 years when both are fused. However, this framework was later challenged due to significant inter-individual and inter-population variability [14].

Further studies across various Indian populations have echoed similar variability. Chandrakanth et al. (2012) studied 118 sterna (67 males and 51 females) in the South Indian population and reported that the earliest age of fusion of the M-S junction was 30 years in females and 35 years in males, while that of sterno-xiphisternal fusion was after the age of 30 years in both sexes. The maximum age at which the M-S junction remains unfused was reported to be 70 years in males and 75 years in females, while the esterno-xiphisternal junction remains unfused latest at the age of 48 years in males and 46 years in females [15]. 

Vijay Kumar et al. (2012) proposed age thresholds based on fusion combinations of the manubrium, body, and xiphoid process and concluded that if the fusion status of manubrium with the body of sternum (M+B) is present, then the age of the individual is above 30 years, and if the fusion status of xiphisternum with the body of sternum (B+X) is present, then the age of the individual is above 35 years. Furthermore, if manubrium, body, and xiphisternum (M+B+X) are fused, then the age of the individual is greater than 40 years. However, they acknowledged that the absence of fusion precludes definitive age estimation [16].

Silajiya et al. (2013) studied 109 sterna of the Indian Gujarati population radiologically and found that the average age of fusion of the M-S junction in males is 50 years and in females is 59 years. The average age of fusion of the sterno-xiphisternal junction in males is 42 years, and in females is 44 years [17].

Chopra et al. (2014) did a similar study in 200 sterna of the Indian population and found that the average age of fusion of the M-S junction in males is 56 years and in females is 61 years. The average age of fusion of the S-X junction in males is 54 years, and in females is 58 years [18].

Both these studies by Silajiya et al. [17] and Chopra et al. [18] observed that females tend to show later fusion than males, a trend also reflected in the present study.

Monum et al. (2017) studied 136 sterna of the Thai male population radiologically and observed that the earliest age at which the M-S and mesosterno-xiphoidal junction fusion occurs is 15 years, while the maximum age of fusion was 81 and 70 years, respectively [1].

Manas R. Sahu et al. (2022) studied 102 sterna at AIIMS Bhubaneswar using the direct inspection method and observed that the earliest age of fusion of the M-S junction is 17 years in females and 24 years in males. The highest fusion rate (69%) was seen between the ages of 51 and 60, but 42% of cases remained unfused even after 60. Thus, the M-S joint is unreliable for age estimation beyond 60. In 17% of cases, the xiphoid process remained cartilaginous past 60 years, with the oldest at 85, though it was almost always fused after age 40 if ossified [19].

Numerous studies have examined the utility of synostosis at the M-S and S-X junctions for age estimation, yet most have highlighted substantial variation in the timing of fusion [20]. The findings of the present research align with previous observations regarding this variability. In our study, the earliest age at which fusion was noted at both the M-S and S-X junctions was 19 years in males and 40 years in females. Conversely, non-fusion was observed up to 82 years in males and 76 years in females at the M-S junction, and up to 85 years in males and 76 years in females at the S-X junction. The fusion of the M-S junction occurred in 23.6% of cases at an average age of 45 years (43 in males, 56 in females). No age correlation was found in males, but a moderate correlation existed in females; ROC analysis in females showed that at age 40 and above, M-S fusion predicts age with 100% sensitivity and 71% specificity, making M-S fusion a reliable age marker for females only. Fusion of the S-X junction was seen in 34.4% of cases at an average age of 47 years (47 in males, 53 in females). A moderate age correlation existed in males; ROC analysis in males showed that at age 34 and above, S-X junction fusion predicts age with 81% sensitivity and 57% specificity. S-X fusion may be useful for age estimation in males.

However, the present study has several limitations. First, its small sample size of 195 sterna limits the generalizability of the results. Second, the uneven male-to-female ratio reduces the reliability of age estimation across sexes, underscoring the need for more balanced representation. Third, since all specimens originated from North India, the findings may not apply to other ethnic or geographic groups. Fourth, the fusion was classified only as complete or absent, without accounting for partial fusion; future research should include intermediate stages to better characterize the progression of sternal junction fusion. Finally, inter- and intra-observer reliability was not formally assessed, which is an important limitation given that evaluation of fusion status may be subject to observer variation.

## Conclusions

Age estimation is essential for constructing an accurate biological profile of an individual in cases where identity is unknown or when limited information is available about the circumstances surrounding their death. Findings from the current study indicate that the sternum offers limited reliability as a marker for age estimation. The fusion pattern at the M-S junction demonstrated considerable variability, thereby reducing its effectiveness in precisely determining chronological age. However, regarding the S-X junction, ROC curve analysis in males revealed that fusion at this site occurs in most men by the age of 34 years (with 81% sensitivity). Still, it is less reliable in identifying younger males (with 57% specificity). Given the small and imbalanced sample, as well as the exclusion of partial fusion stages, the forensic applicability of these findings should be interpreted with caution. Further studies on larger and more diverse populations, with balanced representation of both sexes and inclusion of partial fusion stages, are needed to validate and strengthen these observations.
